# HTLV-1 propels thymic human T cell development in “human immune system” Rag2-/- IL-2R γc-/- Mice

**DOI:** 10.1186/1742-4690-8-S1-A10

**Published:** 2011-06-06

**Authors:** Julien Villaudy, Melanie Wencker, Nicolas Gadot, Jean-Yves Scoazec, Louis Gazzolo, Markus G Manz, Madeleine Duc Dodon

**Affiliations:** 1 Virologie Humaine, INSERM-U758, Ecole Normale Supérieure de Lyon, IFR 128 BioSciences Lyon-Gerland, Lyon, 69364, Cedex 07, France; 2 Anipath, UFR Médecine Lyon-RTH Laennec, Lyon, 69372, Cedex 08, France; 3 Institute for research in Biomedicine (IRB), Bellinzona, 6500, Switzerland; 4 Division of Hematology, University and University Hospital Zürich, Zürich, 8091, Switzerland

## 

Alteration of early haematopoietic development is thought to be responsible for the onset of immature leukemias and lymphomas. We have previously shown that HTLV-1 (Human T cell Leukemia Virus type 1) is able not only to infect immature thymocytes in vitro but also, through Tax expression, to alter the β-selection checkpoint critical for early T cell development. To further clarify the role of the natural HTLV-1 infection on human T-cell development, we developed an in vivo model by transplanting immunocompromised Rag2-/-γc-/- newborn mice with human cord blood CD34+ cells to obtain Human Immune System (HIS) mice. In these mice the development of human T cells in the thymus is fully developed within two months after human cell transplantation. Lethally irradiated HTLV-1 producing cells were then injected into these HIS mice. Herein we observed in the thymus of the infected animals an enlarged population of mature T cells when compared with the mock-infected mice. Furthermore, we noted an increased number of CD4+ cells expressing CD25. Infected animals also developed, several weeks after the infection, pathological features such as splenomegaly, adenopathy, thymomas, lymphomas and leukemias in which predominate human T cells, with a large proportion of CD25+ activated cells. Tax expression especially in the lymphomas and thymomas correlated with an up-regulation of NF-κB regulated genes. Altogether, these results underline that this HIS Rag2-/-γc-/- model might be of great interest to study the leukemogenic process induced by HTLV-1 as well as to validate new therapeutic approaches of ATL.

